# Nicotine Dependence among Rural-Urban Migrants in China

**DOI:** 10.1186/1471-2458-11-296

**Published:** 2011-05-10

**Authors:** Junqing Wu, Tingzhong Yang, Ian RH Rockett, Rui Xing, Sejla Karalic, Yuyan Li, Yufeng Zhang

**Affiliations:** 1 Shanghai Institute of Planned Parenthood Research, WHO Collaborating Center on Human Research on Reproductive Health, Shanghai, 200032, China; 2 Center for Tobacco Control Research, School of Medicine, Zhejiang University, Hangzhou, 310058, China; 3 Injury Control Research Center and Department of Community Medicine, PO Box 9190, West Virginia University, Morgantown, WV 26506-9190, USA; 4 Department of Environmental and Occupational Health, Xinjiang Medical University, Wulumuji, 830054, China

## Abstract

**Background:**

The complex mechanism of nicotine dependency makes it challenging to evaluate dependence or progress towards dependence. The aim of this study was to estimate nicotine dependence levels and identify determinants of dependence among Chinese rural-urban migrants.

**Methods:**

Multi-stage systematic sampling was used to select 4,198 rural-urban migrants aged 18 years or older from three metropolises in China. A structured questionnaire was administered during face-to-face interviews. Nicotine dependence among participants was assessed by means of the six-item Mandarin Chinese Version of the Fagerström Test for Nicotine Dependence (CFTND). Determinants of dependence were analyzed using multivariate analysis of variance (MANOVA).

**Results:**

Among 4,198 participants, estimated current, daily, and occasional smoking rates were 28.3%, 21.2%, and 7.1%, respectively. The CTFND score for the 894 daily smokers was 3.39(SD: 2.32). MANOVA showed that work type, age at first migration, length of migration, and number of cities ever lived were associated with nicotine dependence.

**Conclusion:**

A migratory lifestyle is associated with nicotine dependence. Results could inform the design of tobacco control programs that target Chinese rural-urban migrant workers as a special at-risk population.

## Background

Tobacco smoking and nicotine dependence is a complex syndrome involving physiological, psychological, and behavioral processes [1]. The epidemic of dependence on nicotine harbors enormous adverse consequences for public health. The World Health Organization (WHO) estimated that about 4 million deaths worldwide annually are attributable to tobacco use, a number expected to more than double by 2030 [2]. With more than 80% of these deaths occurring in the less developed world, the epidemic will strike hardest in that subgroup of countries whose rapidly growing economies offer their citizens hope for a much improved standard of living [2]. China, with 20% of the world's people population, experiences almost one million tobacco-related deaths per year [3]. This number is projected to reach 2 million annually by 2025 and 3 million by 2050. Thus, a total of 100 million Chinese will succumb to a smoking-related death in the next 50 years if the current smoking rate persists [4].

China is primarily an agrarian nation, with farmers comprising more than three-quarters of its total population [5]. Rapid modernization and industrialization have triggered massive rural-to-urban migration. Migrants increased from 50 million in 1990 to 121 million by 2000. They were estimated to have reached 160 million by 2010, representing approximately one-quarter of the Chinese working population [5]. While a few studies in other nations have explored nicotine dependence among general migrants [6-8], no studies have focused on rural-urban migrants in China, an especially vulnerable group. Although we have been unable to find any studies that have previously addressed nicotine dependence among Chinese rural-urban migrants, there are studies that examined smoking patterns in this population. Chen and associates found that smoking levels were positively associated with duration of migration and number of cities in which migrants had worked [9], and Yang and associates reported that initiation of daily smoking was associated with history of migration to three cities (adjusted odds ratio (AOR): 1.65) or to four or more cities (AOR: 2.80) versus one city only [10].

We hypothesize that Chinese rural-urban migrants are highly susceptible to nicotine dependence for several reasons. First, their unstable living situations may stimulate smoking [11-13], and secondly their solitude is associated with excess smoking prevalence relative to the general rural population [10]. Thirdly, higher levels of disposable income may elevate their risk both for initiating and increasing tobacco consumption [9].

The complex mechanism of nicotine dependency makes it challenging to evaluate dependence or progress towards dependence [14]. This study is the first to estimate nicotine dependence levels and identify correlates among rural-urban migrant workers who reside in Chinese metropolises. Findings will stimulate and inform the creation, implementation, and evaluation of targeted tobacco use prevention and cessation strategies and programs.

## Methods

### Setting and participants

We employed a stratified multi-stage systematic sampling procedure to recruit participants. In Stage 1, we selected three metropolises based on broad geographical representation. They were Chengdu in southwest China, Shanghai in southeast China, and Beijing in north China. Beijing, the Chinese capital, has a population of approximately 17.4 million [15]. This population includes an estimated 5.4 million migrants. Shanghai is the most populous city in China with 18 million, including approximately 7.1 million migrants [16]. Chengdu is the capital of Sichuan province, and has a population of 11.2 million, including some two million migrants [17]. In Stage 2, we selected one residential district with a high density of rural-urban migrants in each of the three study cities. In Stage 3, we selected as the sampling unit within each district medium-sized or large worksites (operationalized as employing 200 or more workers) that employed migrants. We then randomly sampled 10% of these sites by type [18]: (1) factory (manufacture, machinery, electronics, textile, printing); (2) construction (public and private, transportation and public works infrastructure, and maintenance operations); and (3) service (tourist bureaus, hotels, restaurants, barbershops, beauty salons, bath houses, night clubs, karaoke venues, dance halls, and bars) and commercial sites (markets, street vendors, and small retail shops). In Stage 4, the final stage, we selected as eligible participants from each district cluster of worksite types all rural-urban migrant workers who were ages 18 years or older, held rural "hukou," (that is, were registered permanent residents in a rural area), and had resided in a destination city for at least six months. The number of participants disaggregated by worksite type approximately corresponded to their estimated overall distribution across each district cluster of worksites [9,10,18].

### Data collection

Data were collected by trained interviewers through face-to-face interviews in the households of participants. Various strategies were used to reach migrants at the sample sites. First, we contacted employers at sampling units for permission to conduct the survey on their premises. Upon receipt of this permission, we then contacted recognized group "leaders" who mobilized and encouraged their peers to participate in the study. The study purpose was explained to all eligible participants and verbal consent was obtained from all who elected to participate. The Ethics Committee of the Medical Center at Zhejiang University approved the study protocol. All surveys were administered by local medical professionals, who were trained in research survey methods, and investigators were on-hand to answer questions. Questionnaires were completed by eligible and willing participants and were checked by investigators for completeness. Assistance was provided to participants who experienced difficulty in completion; typically because of an educational deficit. Following completion, all participants were given a token of appreciation (tooth brush and tooth paste; value US$0.50) and asked to provide their names and phone numbers for verification purposes.

### Measures

*Sociodemographic characteristics*: age, gender, education, marital status, work type, monthly income, region of origin, residency status, type of room/residence, and number of cohabiting family members.

*Migration history *age at first migration, length of migration, and number of cities of migration.

*Current smoking status*: current frequency and quantity of smoking. Participants reporting that they used tobacco products, including cigarettes, cigars, rolled cigarettes, and Chinese pipes, were considered smoker*s*. They were divided into daily and occasional smokers. Quantity was calculated by units for pipe smokers and number of cigarettes for the remainder.

*Nicotine Dependence*: assessed using the six-item Mandarin Chinese Version of the Fagerström Test for Nicotine Dependence (CFTND) [13]. Items covered (F1) number of units smoked per day, (F2) time to first cigarette after awakening in the morning, (F3) early excess smoking (smoking status during the first hours) (F4) reluctance to give up the first cigarette in the morning, (F5) difficulty in refraining from smoking in forbidden areas, and (F6) smoking even when ill in bed.

### Data analysis

Data were entered using Microsoft Excel and transferred to SAS. All analyses were conducted using SAS 6.12 Version and in several stages. First, Cronbach's alpha for the CFTND was calculated to test scale reliability. Secondly, we calculated the CFTND total score by summing responses to the six individual items, ranging from 0 (not nicotine dependent) to 10 (most nicotine dependent), and reported scores across demographics. Thirdly, we calculated the arithmetic mean (with standard deviation) to reflect the nicotine dependence level for the sample, and then performed MANOVA to examine the association between nicotine dependence on the one hand and smoking status, demographics, and migration variables on the other. Nicotine dependence was our dependent variable. Our independent variables were age, education, ethnicity, marital status, region of origin, age at first migration, length of migration (years), time spent in city in each year (months), number of cities ever lived in, work type, personal income, type of city residence, and type of smokers. They were all entered into the MANOVA equation. We considered values of p = < 0.05 to be statistically significant.

## Results

### Demographics

A total of 4,825 individuals were identified as eligible participants for our study. Of those, 4,198 were approached and agreed to be interviewed, resulting in a participation rate of 87%. Complete data were reported on the participants due to immediate remedial followup as needed.

Of the 4,198 participants, 53% were male. The great preponderance was under 40 years of age (81%) and earned a personal monthly income of 2,000 RMB or less (81%). Forty-five percent were employed in construction, 32% in the service sector, and 21% in factories. Sixty-two percent of participants had junior school or less education, 47% had worked in the city of destination for five or fewer years, and 25% had worked in two or more cities.

### Smoking prevalence and CFTND scores

The current smoking rate was 28.3%, the daily smoking rate 21.2%, and the occasional smoking rate 7.1%. Among daily smokers (894) the Cronbach alpha for CTFND was 0.67, which indicated acceptable scale reliability. The average number of cigarettes smoked daily was 15.82(SD: 8.09) and 86% of participants reported smoking 10 or more. The total CFND score for all daily smokers was 3.39(SD: 2.32).

### Distribution of CFTND scores for daily smokers

Table 1 shows the mean and standard deviation of CFTND scores for daily smokers pertaining to respondent sociodemographic and migrant characteristics. Scores increased with age, and tended to vary inversely with education. Scores were lowest among the never married than the married and the previously married, and among construction workers relative to other workers. CFTND scores tended to rise with age at first migration, length of migration, and number of cities of migration.

**Table 1:** Distribution of CFTND scores for daily smokers by background characteristics (n = 894)

Group	n	Mean	SD	F	P value
***Age *(in years)**					

< 20	20	2.10	1.80		
		
20-29	236	2.88	2.22		
				11.98	< 0.01
30-39	345	3.35	2.17		
		
40+	293	3.95	2.47		

***Gender***					

male	869	3.41	2.32		
				1.47	> 0.05
female	25	2.84	2.19		

***Educational attainment***					

elementary or lower	134	3.61	2.26		
		
junior high school	483	3.53	2.38		
				3.00	< 0.05
high school	223	3.05	2.23		
		
college or higher	54	3.07	2.07		

***Marital status***					

never married	201	3.00	2.28		
		
married	663	3.50	2.33	3.91	< 0.05
		
divorced/widowed	30	3.73	2.12		

***Work type***					

construction	426	3.62	2.29		
		
factory	288	3.28	2.34	4.62	< 0.05
		
service	180	3.03	2.30		

***Personal income (RMB)/month***					

< 500 RMB	32	3.56	2.14		
		
500-499	131	3.34	2.32		
		
1000-1999	478	3.49	2.33	1.11	> 0.05
		
2000-2999	190	3.10	2.28		
		
> = 3000	63	3.54	2.40		

***Region of origin***					

south-east	227	3.15	2.33		
		
south-west	569	3.51	2.31	2.07	> 0.05
		
Other	98	3.31	2.34		

***Registered residential status***					

rural	726	3.43	2.32		
				1.16	> 0.05
urban	168	3.22	2.28		

***Age at first migration***					

< 20	268	2.95	2.26		
		
20-29	345	3.45	2.28		
				5.67	< 0.01
30-39	224	3.73	2.28		
		
40+	57	3.83	2.61		

***Length of migration (years)***					

< 5	273	3.14	2.22		
		
5-9	289	3.28	2.37		
				4.22	< 0.01
10-14	180	3.51	2.22		
		
15+	152	3.93	2.42		

***Numbers of cities of migration***					

1	263	3.35	2.39		
		
2	304	3.15	2.30		
				3.57	< 0.05
3	146	3.40	2.16		
		
4+	181	3.86	2.33		

***Type of city housing***					

collective dormitory	452	3.35	2.43		
		
room rented by self	251	3.58	2.17		
		
room rented by self and other family members	133	3.37	2.29	0.97	> 0.05
		
room owned by self	44	3.20	2.14		
		
other	17	2.65	2.00		

**Nicotine dependence and associated factors**

Table 2  shows the results of the MANOVA analyses of CFTND scores among daily smokers. Score were higher among construction workers than factory and service workers. Age at first migration, length of migration, and number of cities of migration were all associated with nicotine dependence.

**Table 2:** Table 2 shows the results of the MANOVA analyses of CFTND scores among daily smokers

Group	N	Mean	SD	F	P value
***Work type***					

construction	426	3.62A	2.29		
		
factory	288	3.28B	2.34	11.64	< 0.01
		
service	180	3.03B	2.30		

***Age at first migration***					

< 20	268	2.95A	2.26		
		
20-29	345	3.45AB	2.28		
				7.29	< 0.01
30-39	224	3.73B	2.28		
		
40+	57	3.83B	2.61		

***Length of migration (years)***					

< 5	273	3.14A	2.22		
		
5-9	289	3.28A	2.37		
				16.18	< 0.01
10-14	180	3.51A	2.22		
		
15+	152	3.93B	2.42		

***Numbers of cities of migration***					

1	263	3.35A	2.39		
		
2	304	3.15AB	2.30	3.10	< 0.05
		
3	146	3.40ABC	2.16		
		
4+	181	3.86C	2.33		

Score were higher among construction workers than factory and service workers. Age at first migration, length of migration, and number of cities of migration were all associated with nicotine dependence. MANOVA of factors associated with nicotine dependence among daily smokers*

*Shared letters across groups indicate non-significant intergroup differences and different letters significant differences.

## Discussion

A better understanding of nicotine dependence will contribute to the design of evidence-based treatment strategies for nicotine dependence. It could be imprudent to assume that patterns of tobacco smoking and nicotine dependence rural-urban migrants mirror those observed in general population. This study is the first to evaluate nicotine dependence in Chinese rural-urban migrants. We determined that the Chinese version of CFTND was acceptably reliable for estimating and analyzing dependence in our study population of adult rural-urban workers. The total CFTND score of 3.39 (3.24-3.54) was lower than that reported in some studies [19,20], but higher than that in others [21]. However, there is no significant difference between scores for our urban population 3.47(3.35-3.62) [13]. By virtue of their sociodemographics and the impact of the migration process, we posit that migrant workers form a population quite distinct from general rural and urban populations. Thus, we acknowledge difficulty in making direct inter-population comparisons. Whereas many studies reported significant associations between nicotine dependence and general demographics [13,19-23] we found none. On the other hand, we did find associations with some specific aspects of migration.

With respect to work type, we found that nicotine dependence peaked among migrant daily smokers who engaged in construction. Almost half of the rural-urban migrants are in that occupation [9,10]. Construction is burdensome and hazardous, but the salary is very low and remuneration is often delayed or denied [18]. Construction workers have the lowest socioeconomic status among the migrants. In exacerbating stress, employment in construction makes migrant workers very prone to nicotine dependence [18]. The association between age at first migration and degree of nicotine dependence likely reflects the fact that younger migrants adapt more poorly to urban life than older counterparts - leading to additional stress, increased susceptibility to smoking and nicotine dependence [9]. We found that number of cities of migration was associated with nicotine dependence, consistent with findings on smoking behavior from other studies [9,10]. This may be because unstable living situations make people more prone to heavy smoking [11-13]. The association we found between length of migration and nicotine dependence probably stems from the cumulative stressful effects of adverse migrant living and working circumstances [11-13].

Yang and associates reported that 38% of rural urban migrants first migrated before 20 years of age, 60% moved to two or more cities, and 79% resided in their destination cities for 3 years or longer [10]. Chen and associates reported that 57% of rural-urban migrants had moved to two or more cities and slightly less than two-thirds had three or more years of migratory experience [9]. Those findings and ours collectively show that the relationship between migration history and nicotine dependence represents a serious health problem in China.

Some special prevention initiatives, which address nicotine dependence, should target rural-urban migrants. Both government and local health authorities need to formulate tobacco control policies and design, implement, and evaluate prevention strategies and programs to confront the epidemic of nicotine dependence in the migrant population. Prevention needs to be an integral feature of community healthcare programs, and different types of health services should be provided to the migrant population. For example, a community-based tobacco control health education curriculum should be developed to target this group through schools, workplaces, and households. Its content should include identification of relevant risk factors for nicotine dependence. Moreover, special counseling should be part of existing community health services to enable rural-urban migrants to eschew or cease smoking as pertinent.

There are a number of study limitations. These limitations relate to sampling, subject heterogeneity, and research design. Sampling is very complex issue, and it was infeasible for us to obtain a truly national sample of rural-urban migrants. It is while value to be explored fourth. Moreover, we were unable to capture the total complexity of rural-urban migrant existence, including issues of unemployment and family employment. Only about three percent of rural-urban migrants are unemployed [24], and less than two percent have family employment [25]. Another study limitation was that our cross-sectional design precluded causal inference.

## Conclusion

Our study suggests that the migratory process and urban maladaptation induce nicotine dependence among Chinese rural-urban migrants. Findings provide justification for designing and implementing appropriate tobacco use prevention and cessation strategies and programs.

## Competing interests

The authors declare that they have no competing interests.

## Authors' contributions

TY conceived the study design, conceptualized the ideas, and supervised the data management and analyses. JW, RX, YL, and YZ conducted the data collection. IR provided technical support for the data analysis and participated in drafting and revising the manuscript. SK assisted in the preparation of the first draft. All authors read and approved the manuscript.

## Pre-publication history

The pre-publication history for this paper can be accessed here:

http://www.biomedcentral.com/1471-2458/11/296/prepub
